# The Hôtel-Dieu MRI Classification of Uterosacral Ligament Involvement in Endometriosis: A Pictorial Guide to Clinical Use

**DOI:** 10.3390/diagnostics15121508

**Published:** 2025-06-13

**Authors:** Siegfried Hélage, Claudia Laponche, Margaux Homps, Jonathan Dong, Lucas Rivière, Frédéric Préaux, Pierre-Alexandre Just, Nizar Aflak, Jean-Noël Buy, Élisabeth Dion

**Affiliations:** 1 Department of Radiology, Hôtel-Dieu de Paris (AP-HP), 1 place du Parvis Notre-Dame, 75004 Paris, France; 2 Biomedical Imaging Group, École Polytechnique Fédérale de Lausanne (EPFL), 1015 Lausanne, Switzerland; 3 Léonard de Vinci Medical Imaging, 43 rue Cortambert, 75016 Paris, France; 4 Department of Pathology, Hôpital Cochin (AP-HP), 27 rue du Faubourg Saint-Jacques, 75014 Paris, France; 5 Department of Gynecological Surgery, Hôpital Beaujon (AP-HP), 100 boulevard du Général Leclerc, 92110 Clichy, France

**Keywords:** deep infiltrating endometriosis, DIE, uterosacral ligament, USL, MRI classification, Hôtel-Dieu, HTD, microRNA, miRNA

## Abstract

**Objectives:** Endometriosis is a common gynecologic condition characterized by the presence of endometrial-like tissue outside the uterus, often leading to pelvic pain and infertility. Diagnosis is frequently delayed, with prolonged diagnostic wandering that could be improved through enhanced first-line radiologic assessment. The uterosacral ligament (USL) is the most frequent site of deep infiltrating endometriosis (DIE). The Hôtel-Dieu (HTD) MRI classification, published in 2024, offers a structured framework for evaluating USL involvement by correlating MRI findings with the diagnostic certainty of endometriosis. **Key Findings:** This pictorial essay provides a practical guide for applying the HTD MRI classification, presenting key imaging criteria with illustrative examples for each USL type. The classification distinguishes between “linear” and “nodular” USL lesions, with implications for diagnostic confidence. “Nodular“ types demonstrate a 100% positive predictive value (PPV), while “linear“ types may yield higher false positive rates (FPR). The HTD MRI classification may also be complemented by innovative biomarker testing, such as microRNA signatures, especially in cases with “linear“ USL involvement. **Conclusions:** By standardizing the assessment of USL lesions, the HTD MRI classification enhances diagnostic accuracy, improves MRI reproducibility, and supports earlier identification of endometriosis in first-line settings. Its integration into radiologic workflows can contribute to reduced diagnostic delays. **Implications for practice:** The HTD MRI classification is a valuable screening tool for first-line radiologists and clinicians. Incorporating it into routine pelvic MRI interpretations may streamline diagnostic pathways, promote consistency across readers, and guide additional testing strategies, such as microRNA assays, for cases where MRI alone is less definitive.

## 1. Introduction

Endometriosis is a chronic gynecological condition characterized by the presence of both endometrial glands and stroma outside the uterine cavity [1]. This condition affects approximately 10% of women of reproductive age, 40% of women with infertility, and up to 70% of women with chronic pelvic pain [2]. Symptoms vary significantly in nature and intensity, the most common being dysmenorrhea, deep dyspareunia, dysuria, dyschezia, infertility, and chronic pelvic pain. Some patients may remain asymptomatic [3].

Endometriosis is classified into three phenotypes based on the location of ectopic endometrial-like tissue: peritoneal (i.e., superficial), ovarian (i.e., endometrioma), and deep infiltrating endometriosis (DIE), which is defined as lesions penetrating 5 mm or more beneath the peritoneal surface [4,5]. DIE primarily affects the uterosacral ligaments (USLs), torus uterinus, rectum, rectosigmoid junction, bladder, ureters (potentially causing upstream hydronephrosis), and the sacro-recto-genital septum (a.k.a. Delbet sagittal fascia), which encloses the inferior hypogastric plexus [6].

Diagnosing endometriosis remains challenging. Gynecological clinical examinations and transvaginal pelvic ultrasounds have limited accuracy, particularly for superficial peritoneal lesions and USL involvement [3,7]. The gold standard is laparoscopy with histological confirmation, but its invasive nature poses a significant drawback [8]. Despite some subjectivity in image interpretation, MRI is currently the most effective non-invasive tool for diagnosing DIE and mapping deep lesions, offering high-resolution and detailed anatomical visualization [9,10].

The USLs, which are the primary posterior support structures of the uterus, can be identified on T2-weighted imaging (T2WI) in axial and sagittal planes, appearing outlined by the pelvic fatty connective tissue. They extend from the torus uterinus to the presacral fascia at the S2-S3 level, though variations in their origin and termination are documented. Umek et al. [11] described anterior attachments to the torus uterinus (33%), to the cervix and posterior vaginal fornix (63%), or exclusively to the posterior vaginal fornix (4%). Posteriorly, the USLs may be inserted on the sacrospinous ligament/coccygeus muscle complex (82%), the sacrum (7%), or less commonly on the piriformis muscle, sciatic foramen, or ischial spine (11%).

USL involvement is observed in 70% of patients with DIE [12,13], making it the second most frequent site of endometriosis after ovarian involvement [9,14]. On MRI, USL endometriosis typically appears as hypointense nodules on T2WI, or as unilateral or bilateral thickening of these ligaments with regular or irregular margins [13,15]. Lesions are often located near the torus uterinus or along the proximal third of the USL, corresponding to the segment within 2 cm of the torus, which aligns anatomically with the posterior vaginal fornix [16].

Improving the accuracy of MRI in diagnosing DIE requires specific strategies. Since the USLs are frequently and early affected in DIE, the development of a classification system for USL appearance on MRI, linked to the diagnostic certainty of endometriosis, was considered essential. Assessing USL lesions can be challenging, particularly for non-expert radiologists, and significant variability exists in evaluating USL involvement [9,17,18]. To address this subjectivity, the Hôtel-Dieu (HTD) MRI classification system was proposed and evaluated in a 2024 study that compared MRI findings with histological analyses in symptomatic women [19].

The HTD MRI classification is a semi-objective system that stratifies the likelihood of DIE in women with suggestive symptoms. This classification employs partially subjective, minimally or non-quantitative criteria to categorize each USL. The system includes two main categories (linear and nodular) and six types: types 1, 2, 3A, and 3B in the linear category (“L-category”), and types 4, 5A, 5B, and 6 in the nodular category (“N-category”). The study found that nodular types had a 100% positive predictive value (PPV) and 0% false positive rate (FPR) for DIE in symptomatic women. Linear types had PPVs of 88% (type 1, FPR = 11%), 83% (type 2, FPR = 16%), 75% (type 3A, FPR = 25%), and 80% (type 3B, FPR = 20%) [19].

By distinguishing between linear and nodular USLs, the HTD MRI classification addresses the limitations of MRI specificity reported in the literature, which show an overall false positive rate of 10% [20], increasing to 23% for DIE when compared with surgical findings [21]. Linear-type USLs, in particular, may be observed in asymptomatic women [11].

This pictorial essay aims to present the HTD MRI classification through annotated images illustrating each USL type. It provides a series of key visual references for radiologists and clinicians, facilitating the application of this semi-objective classification. By improving the interpretation of MRI findings, this atlas aims to reduce diagnostic delays and optimize the management of symptomatic women.

## 2. The HTD MRI Classification

### 2.1. The “L-Category”: Linear Types of USLs (1, 2, 3A and 3B)

#### 2.1.1. HTD Type 1 USL (PPV = 88%; FPR = 11%)

**Figure 1:**
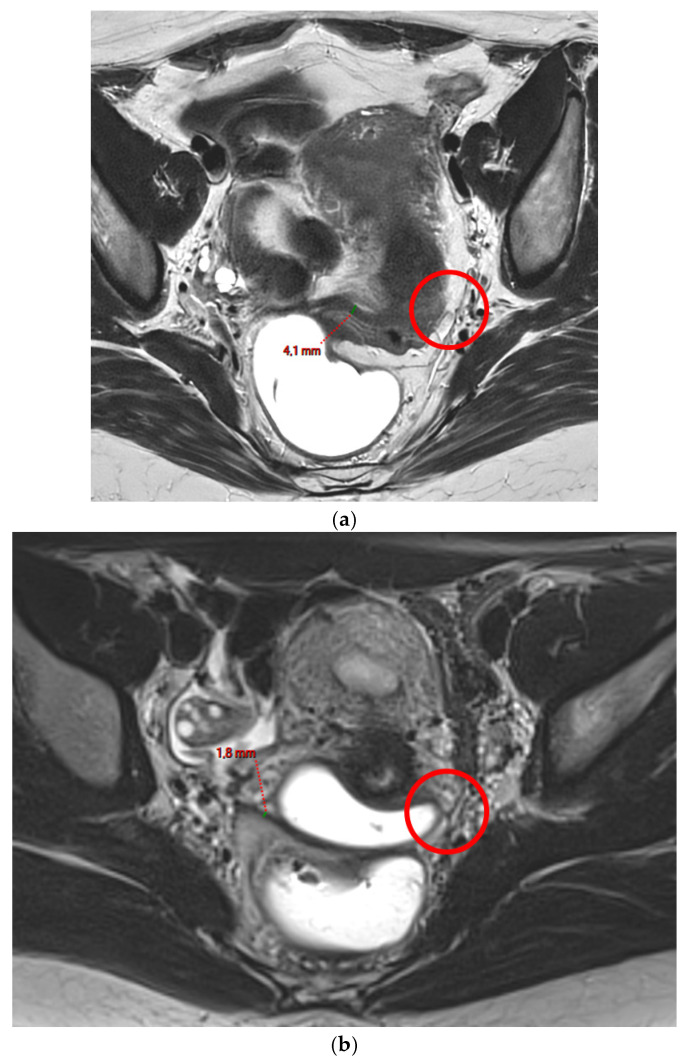
Pelvic MRI scans of two patients with non-visible left USLs (HTD type 1). (**a**) Axial T2WI shows a visible and measurable right USL but a non-visible left USL (*red circle*). (**b**) Axial T2WI shows a visible and measurable right USL but a non-visible left USL (*red circle*).

A type 1 USL (Figure 1) is not visible on MRI scans.

#### 2.1.2. HTD Type 2 USL (PPV = 83%; FPR = 16%)

**Figure 2:**
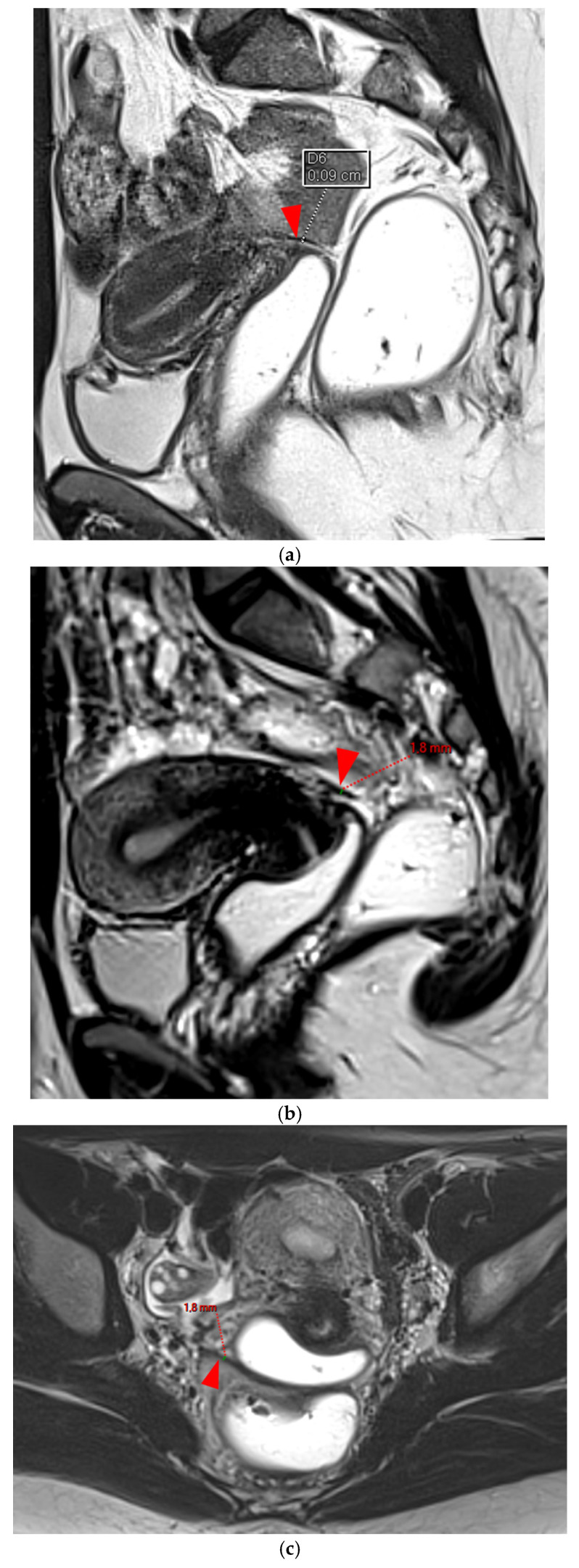

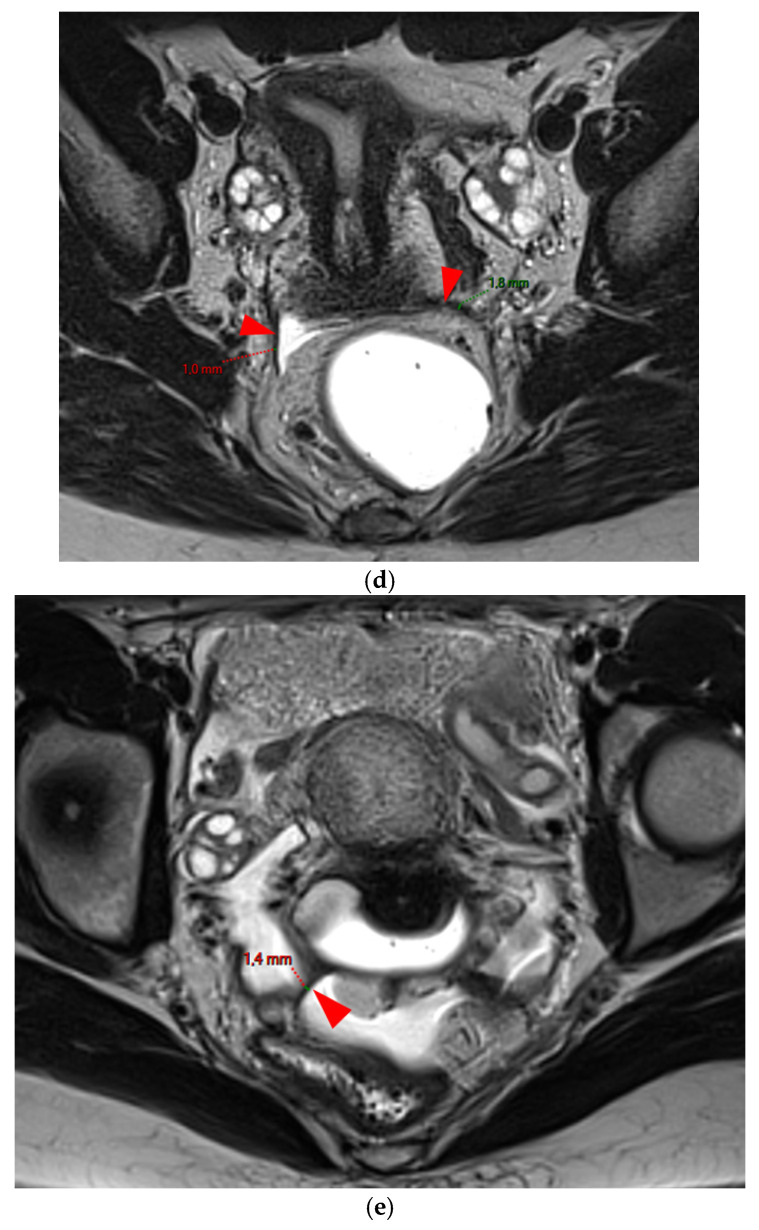
Pelvic MRI scans of five patients with visible but thin (≤2 mm), smooth, and regular USLs (HTD type 2). (**a**) Sagittal T2WI: right USL (*arrowhead*). (**b**) Sagittal T2WI: right USL (*arrowhead*). (**c**) Axial T2WI: right USL (*arrowhead*). (**d**) Axial T2WI: left and right USLs (*arrowheads*). (**e**) Axial T2WI: right USL (*arrowhead*).

A type 2 USL (Figure 2) is visible on MRI scans as a curvilinear T2 hypointense structure that mainly originates from the posterior uterine wall at the uterocervical junction, where the torus uterinus is located, and courses dorsocranially toward the sacrum. A type 2 USL is either visible but not measurable due to its minimal thickness (i.e., ≤1 mm), or visible and measurable but thin (i.e., between 1 mm and 2 mm, inclusive of 2 mm). It appears smooth with regular margins and often displays a longitudinally tapering shape.

#### 2.1.3. HTD Type 3 USL (PPV Between 75% and 80%; FPR Between 20% and 25%)

A type 3 USL appears as thickened (i.e., >2 mm), and can be classified either as type 3A (PPV = 75%; FPR = 25%) or as type 3B (PPV = 80%; FPR = 20%).

##### HTD Type 3A USL

**Figure 3:**
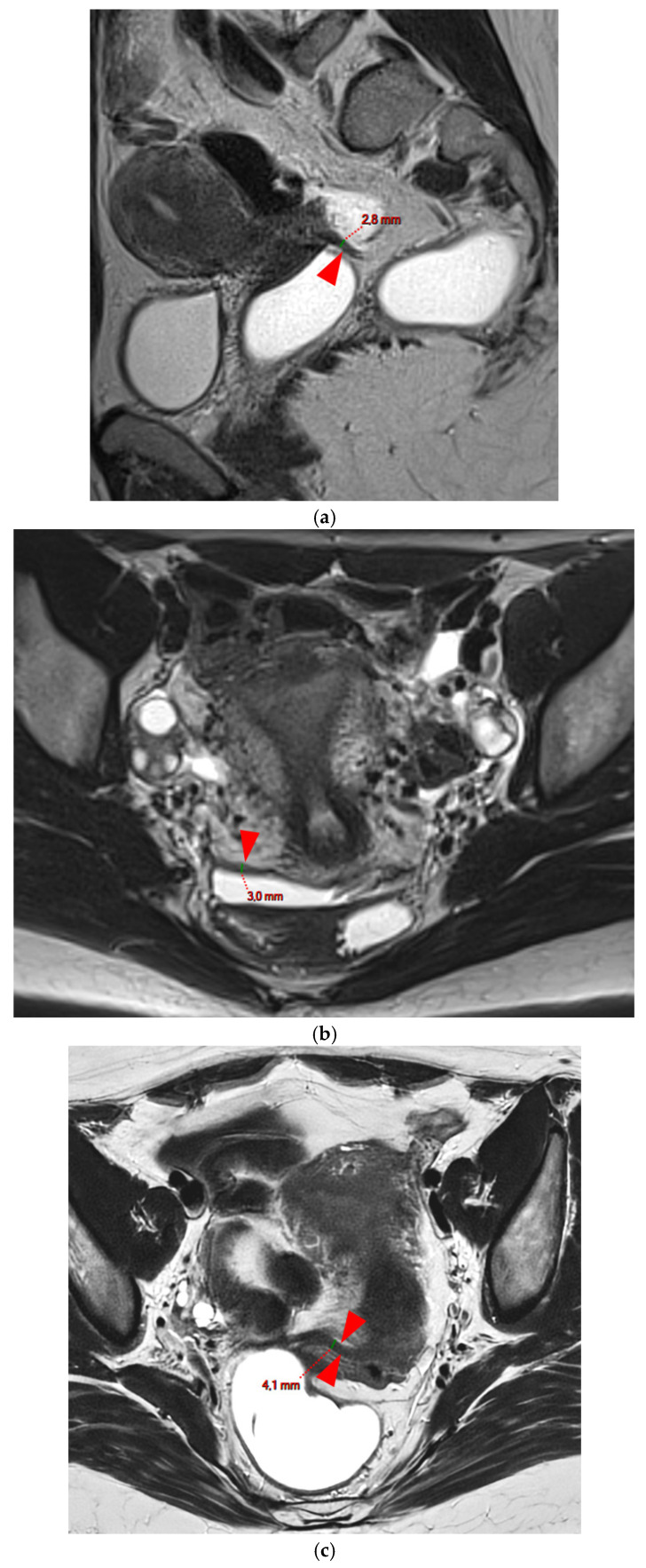
Pelvic MRI scans of three patients with thick (>2 mm), smooth, and regular USLs (HTD type 3A). (**a**) Sagittal T2WI: a thick (2.8 mm), smooth, tapering-shaped left USL (*arrowhead*). (**b**) Axial T2WI: regularly thickened (3 mm) right USL with a smooth surface (*arrowhead*). (**c**) Axial T2WI: regularly thickened (4.1 mm) right USL with a smooth surface (*arrowheads*).

A type 3A USL (Figure 3) maintains a smooth appearance with regular margins and often retains a tapering shape despite its thickening.

##### HTD Type 3B USL

**Figure 4:**
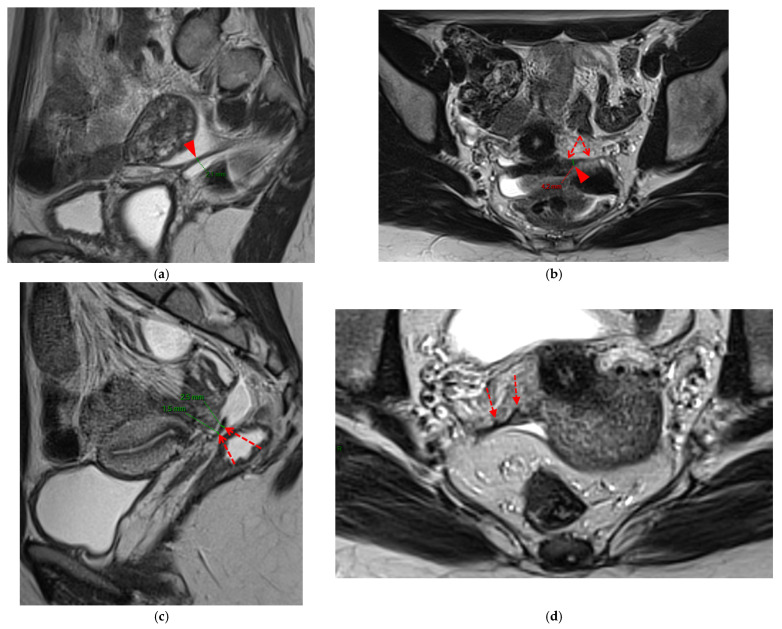

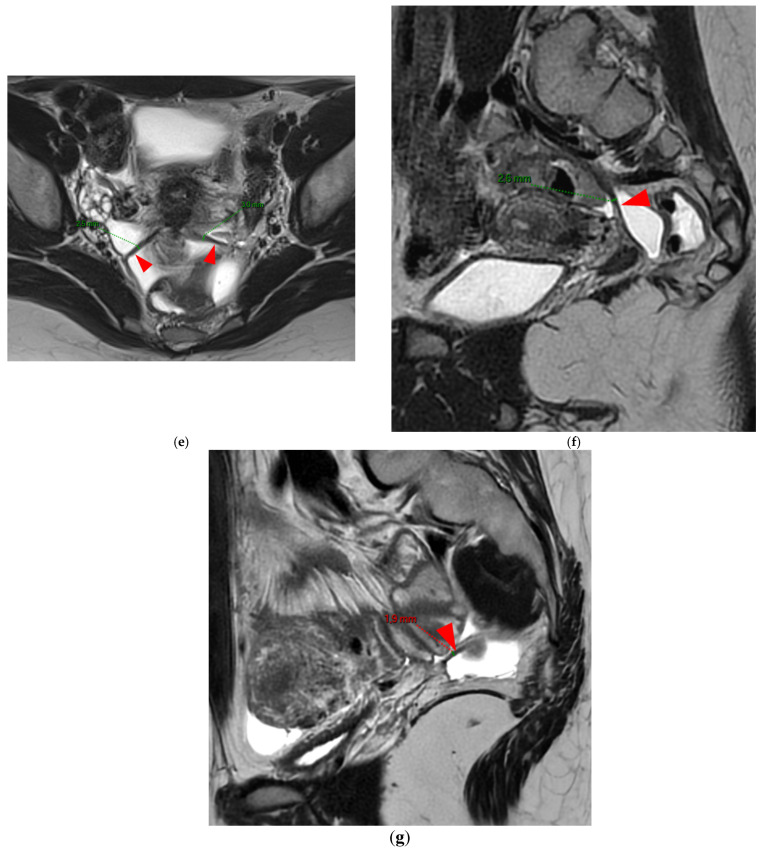
Pelvic MRI scans of seven patients with HTD type 3B USLs. (**a**) Sagittal T2WI: a thickened (2.1 mm) and stiffened right USL (*arrowhead*) with “bowstringing”. (**b**) Axial T2WI: a caliber disparity (*dashed arrows*) with focal thickening (4.2 mm) of the left proximal USL (*arrowhead*). (**c**) Sagittal T2WI: a caliber disparity (*dashed arrows*) with focal thickening (2.5 mm) of the right distal USL. (**d**) Axial T2WI: a right USL with a notched and irregular surface (*dashed arrows*). (**e**) Axial T2WI: thickened and stiffened left (3 mm) and right (2.5 mm) USLs with “bowstringing” of both USLs (*arrowheads*). (**f**) Sagittal T2WI: a thickened (2.6 mm) right USL with a stiffened appearance characterized by a steep vertical orientation (*arrowhead*). (**g**) Sagittal T2WI: the right USL appears thin (1.9 mm) but stiffened (*arrowhead*), exhibiting “bowstringing”. These findings led to its reclassification from type 2 to type 3B.

A type 3B USL (Figure 4), in addition to being thickened, has a notched surface with slightly irregular margins, or a caliber disparity with focal thickening, or appears “stiffened”, which means it loses its curvilinearity to exhibit a steep vertical orientation in the sagittal plane or a “bowstringing” of the USL in the sagittal or axial planes. Occasionally, a USL might appear thin but “stiffened”, which would upgrade it from a type 2 to a type 3B.

### 2.2. The “N-Category”: Hemorrhagic or Nodular Types of USLs (4, 5A, 5B and 6)

#### 2.2.1. HTD Type 4 USL (PPV = 100%; FPR = 0%)

**Figure 5:**
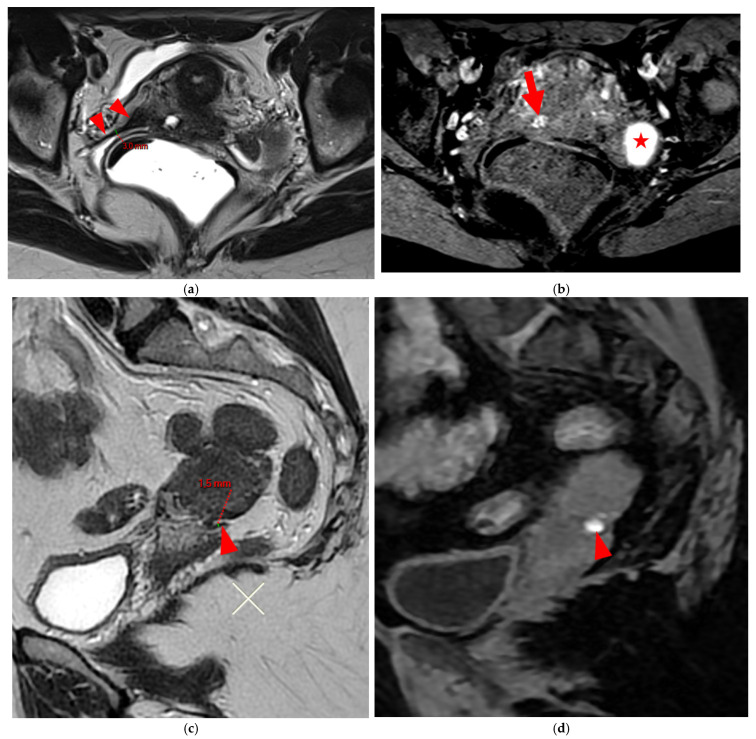
Pelvic MRI scans of two patients with HTD type 4 USLs. (**a**,**b**) Patient 1—(**a**) Axial T2WI: a focally thickened right proximal USL (*arrowhead*) with caliber disparity, initially classified as a type 3B USL. (**b**) Axial fat-suppressed T1WI: a hyperintense hemorrhagic spot (*arrowhead*) within this thickened right proximal USL, ultimately reclassifying it as a type 4 USL. Note the typical hyperintense right ovarian endometrioma (*red star*). (**c**,**d**) Patient 2—(**c**) Sagittal T2WI: a visible but thin (1.5 mm) left USL (*arrowhead*), initially classified as a type 2 USL. (**d**) Sagittal fat-suppressed T1WI: a hyperintense hemorrhagic spot (*arrowhead*) at the origin of this USL, ultimately reclassifying it as a type 4 USL.

A type 4 USL (Figure 5) contains hemorrhagic implants, visible on MRI as hyperintense spots on fat-suppressed T1WI.

#### 2.2.2. HTD Type 5 USL (PPV = 100%; FPR = 0%)

A type 5 USL appears nodular and can be classified either as type 5A or type 5B.

##### HTD Type 5A USL

**Figure 6:**
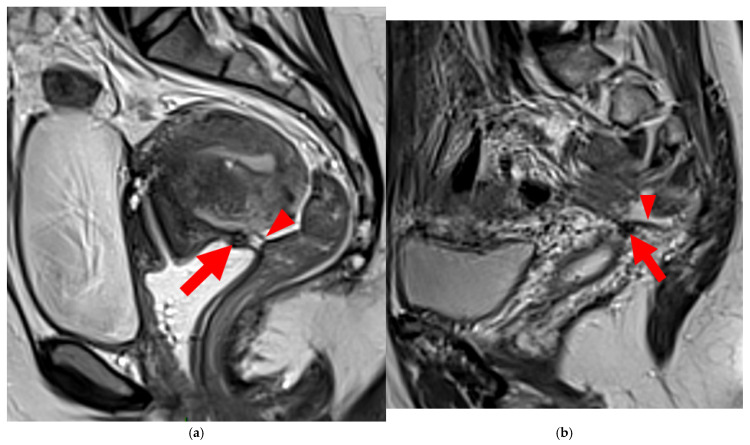
Pelvic MRI scans of two patients with HTD type 5A USLs. (**a**,**b**) Sagittal T2WI: nodularity with regular margins (*arrows*) within the right USL (*arrowheads*).

A type 5A USL (Figure 6) is nodular with a smooth contour.

##### HTD Type 5B USL

**Figure 7:**
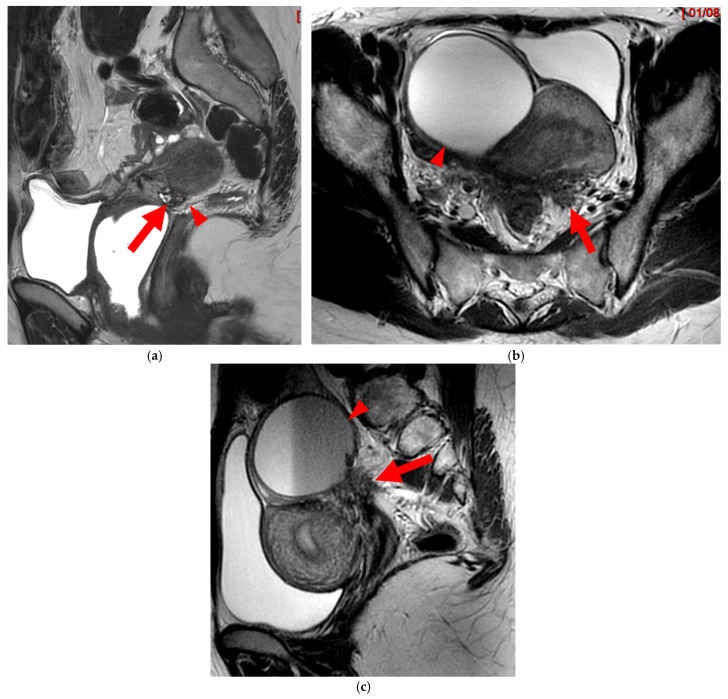
Pelvic MRI scans of two patients with HTD type 5B USLs. (**a**) Patient 1: sagittal T2WI shows a microcystic nodule (*arrow*) within the origin of the right USL (*arrowhead*). (**b**,**c**) Patient 2: axial (**b**) and sagittal (**c**) T2WI show a nodular left USL with spiculated margins (*arrow*) and a right ovarian endometrioma (*arrowhead*).

A type 5B USL (Figure 7) is nodular with spiculated margins. A type 5B USL may also display an isolated nodule with microcystic content.

#### 2.2.3. HTD Type 6 USL (PPV = 100%; FPR = 0%)

**Figure 8:**
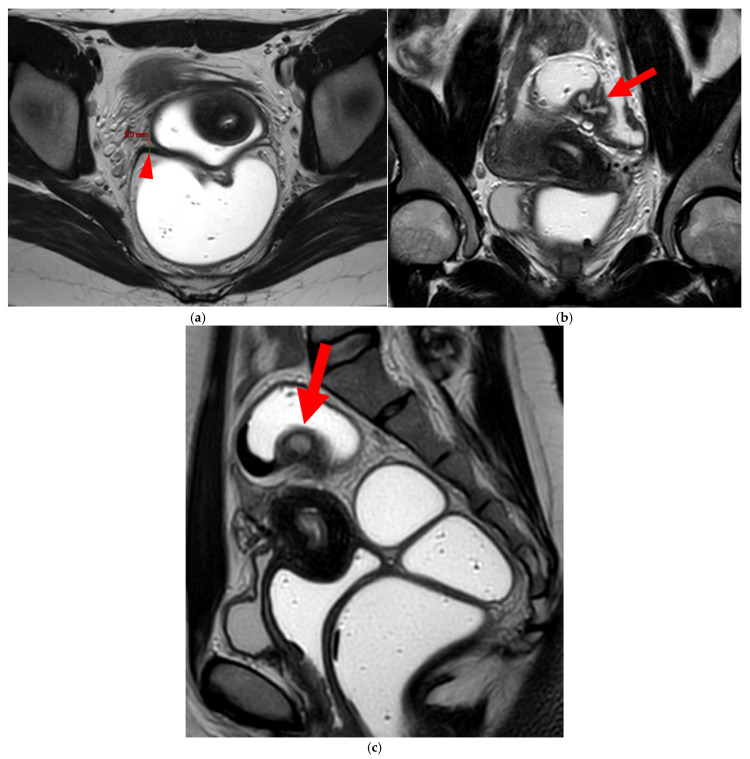
Pelvic MRI scan of a patient with HTD type 6 USLs. (**a**) Axial T2WI shows a thick (3 mm) right USL (*arrowhead*) with regular margins, initially classified as a type 3A USL; the left USL is not visible (type 1). (**b**,**c**) Coronal (**b**) and sagittal (**c**) T2WI show sigmoid colon wall infiltration appearing as a “medallion-shaped” lesion (*arrows*). The presence of “visceral” involvement of the digestive tract leads to the reclassification of these USLs as type 6.

**Figure 9:**
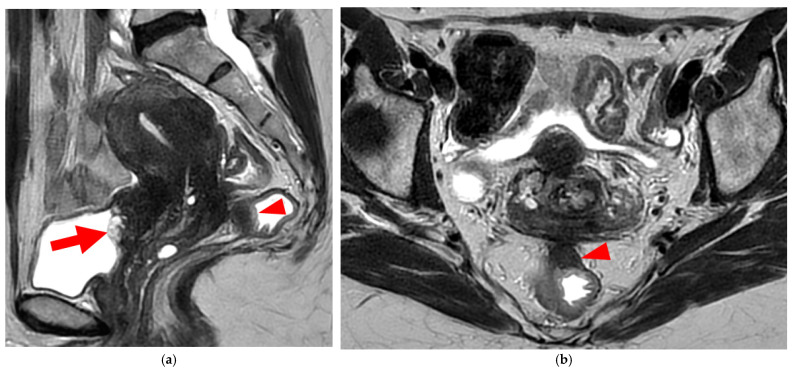

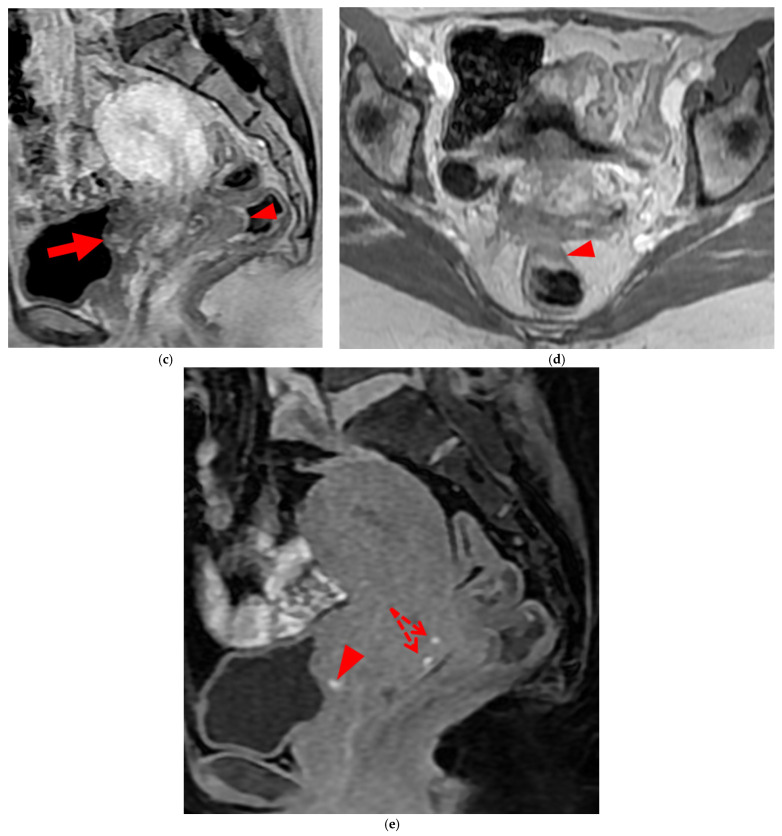
Pelvic MRI scan of a patient with HTD type 6 USLs. (**a**–**d**) Sagittal (**a**) and axial (**b**) T2WI, sagittal (**c**) and axial (**d**) contrast-enhanced T1WI demonstrate involvement of the posterior bladder wall (*arrows*) and rectal wall infiltration appearing as a “medallion-shaped” lesion (*arrowheads*). (**e**) Sagittal fat-suppressed T1WI shows two hyperintense hemorrhagic spots at the origin of the USLs (*dashed arrows*) and another within the bladder lesion (*arrowhead*). The presence of “visceral” involvement of both the digestive and urinary tracts results in reclassifying these type 4 USLs as type 6.

**Figure 10:**
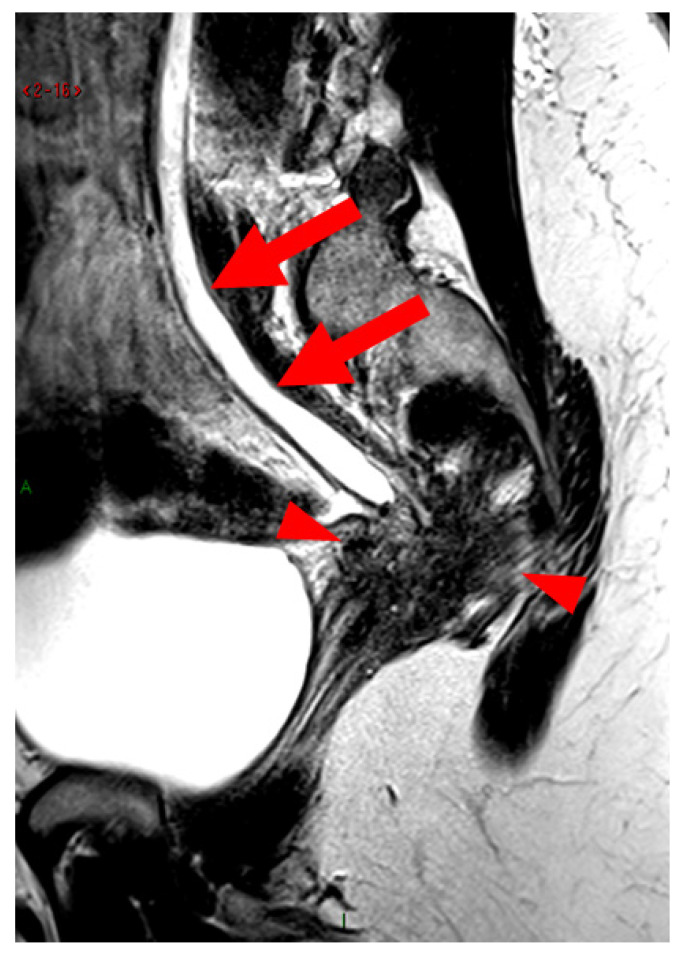
Pelvic MRI scan of a patient with HTD type 6 USLs. Sagittal T2WI demonstrates nodulospicular infiltration of the sacro-recto-genital septum, with the endometriotic lesion delineating the structure of the inferior hypogastric plexus (*arrowheads*). Note that the lesion also involves the distal ureter, causing upstream ureteral dilation (*arrows*).

**Figure 11:**
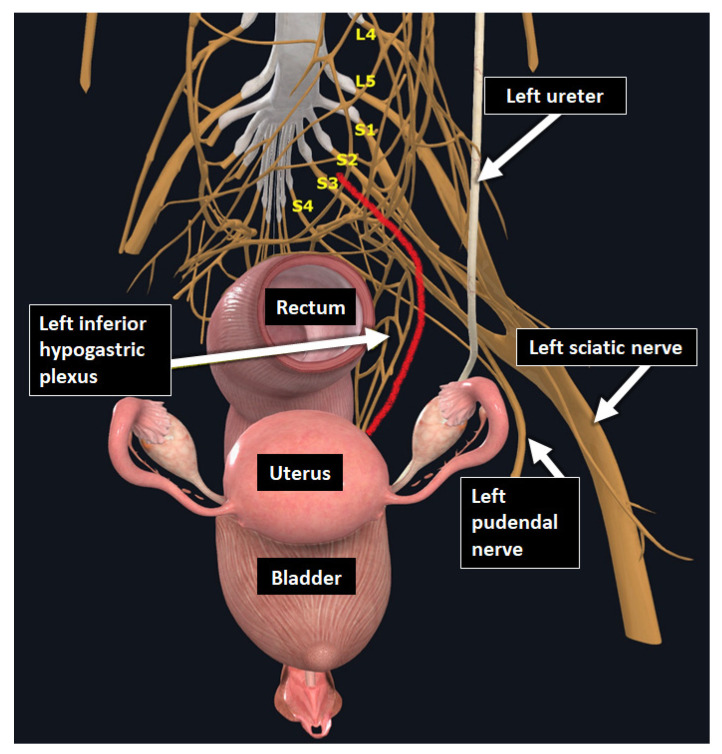
3D schematic anterior view of the female pelvis showing the proximity between the USLs and pelvic nerve structures. For instance, the left USL (*red curved line*) can be considered the roof of the sacro-recto-genital septum, which contains the nerve fibers of the inferior hypogastric plexus aligned with the medial part of the broad ligament. The distal termination of the USL lies near the sacral roots that form the sacral plexus, the main trunk of which is the sciatic nerve, traveling along the lateral part of the broad ligament.

**Figure 12:**
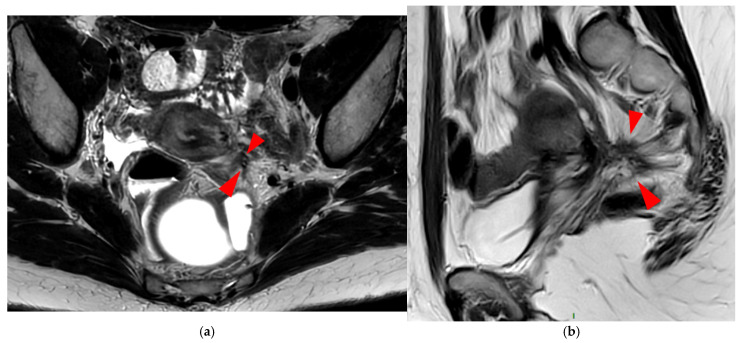
Pelvic MRI scan of a patient with a HTD type 6 left USL. (**a**,**b**) Axial (**a**) and sagittal (**b**) T2WI show a spiculated nodular hypointense lesion (*arrowheads*) extensively involving the left inferior hypogastric plexus contained within the sacro-recto-genital septum, as well as its afferent and efferent nerve fibers.

**Figure 13:**
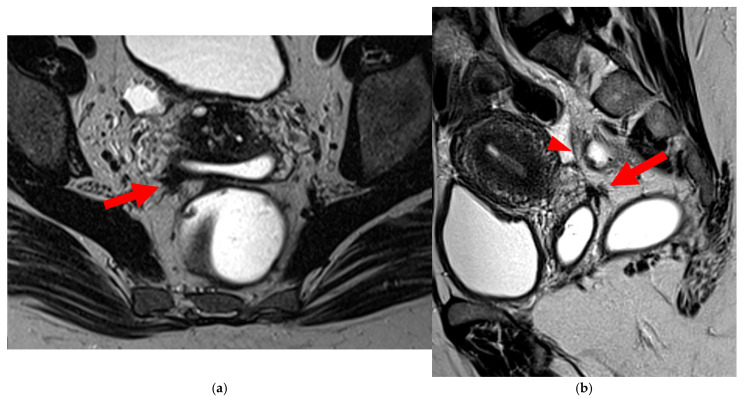
Pelvic MRI scan of a patient with a HTD type 6 right USL. (**a**,**b**) Axial (**a**) and sagittal (**b**) T2WI show a spiculated nodular hypointense lesion (*arrows*) located in the right cardinal ligament (a.k.a. Mackenrodt ligament), affecting the uterovaginal nerve plexus, a component of the inferior hypogastric plexus. Note the steep vertical orientation of the right proximal USL (*arrowhead*), which is reclassified from type 3B to type 6 due to this so-called “visceral” involvement.

**Figure 14:**
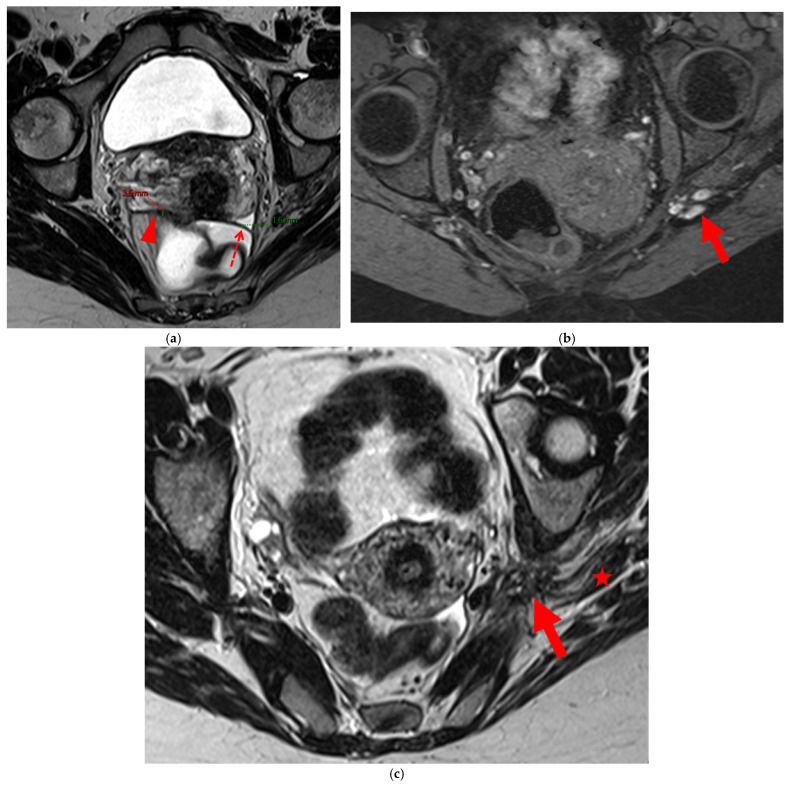
Pelvic MRI scan of a patient with a HTD type 6 USL. (**a**) Axial T2WI shows a visible but thin (1.6 mm) left USL (*dashed arrow*) and a thick (3.5 mm) but smooth right USL (*arrowhead*), initially classified as a type 2 left USL and a type 3A right USL. (**b**) Axial fat-suppressed T1WI reveals hyperintense hemorrhagic spots within the left sciatic nerve (*arrow*). (**c**) Axial T2WI demonstrates spiculated nodularity within the left sciatic nerve (*arrow*), leading to reclassification as type 6 left USL due to this so-called “visceral” nerve involvement. Note the neurogenic amyotrophy (*red star*) of the left piriformis and gluteal muscles, including the gluteus maximus, gluteus medius, and gluteus minimus.

A type 6 USL is associated with adjacent pelvic “visceral” involvement in a broad sense. It most commonly affects the digestive tract (Figure 8 and Figure 9), with the rectum and rectosigmoid junction wall thickening: in this case, the lesion often appears as a “medallion-shaped” protrusion into the lumen. Less frequently, the urinary tract is affected (Figure 9 and Figure 10), involving the muscular layer of the bladder or even the distal ureter at the level of the common iliac artery, with stenosis potentially leading to upstream hydronephrosis. More rarely, as USLs are also close to nearby pelvic nerve structures (Figure 11), contiguous involvement of the inferior hypogastric plexus located in the sacro-recto-genital septum (a.k.a. Delbet sagittal fascia) beneath the distal two-thirds of the USL, or exceptional involvement of the sciatic nerve adjacent to the pelvic wall, is possible (Figure 12, Figure 13 and Figure 14).

### 2.3. Special Cases

#### 2.3.1. “Kissing Ovaries”: A Form Straddling Between Type 5B and Type 6 USLs

Besides endometriomas, another type of ovarian involvement in DIE includes adhesions that cause the ovaries to retract medially across the midline, behind the uterus, into the pouch of Douglas. When the ovaries are displaced medially and are in close proximity, they are commonly referred to as “kissing ovaries”. Additionally, medialized ovaries on preoperative imaging expose the digestive tract to a 20% risk of involvement [22]. In our experience, the injection of a contrast agent is useful when there is doubt about an endometriotic rectosigmoid invasive lesion on T2WI, especially if a rectal filling was not performed. A markedly enhancing mucosa, which outlines rectosigmoid local wall thickening on post-contrast T1WI without fat suppression (forming a “medallion-shaped” lesion), is a valuable aid to diagnosis.

**Figure 15:**
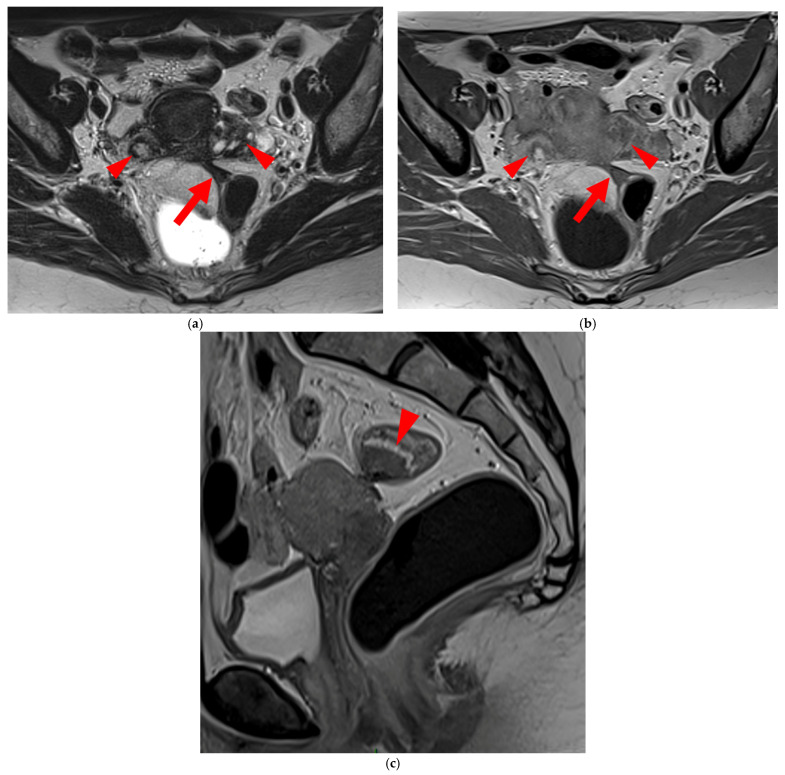
Pelvic MRI scan of a patient with a “kissing ovaries” sign and rectal involvement (i.e., HTD type 6 USLs). (**a**,**b**) Axial T2WI (**a**) and contrast-enhanced T1WI (**b**) show both ovaries in close proximity (“kissing ovaries” sign) (*arrowheads*) and rectosigmoid wall infiltration (*arrows*). (**c**) Sagittal contrast-enhanced T1WI shows rectosigmoid wall infiltration, appearing as a “medallion-shaped” lesion outlined by markedly enhancing mucosa (*arrowhead*).

In the HTD MRI classification of USLs, the “kissing ovaries” sign is considered a form between types 5B and 6. In the case of concomitant rectal involvement, the USL is classified as type 6 (Figure 15); otherwise, it is classified as type 5B (comparable to a nodule with spiculated margins).

#### 2.3.2. Superficial Endometriosis

**Figure 16:**
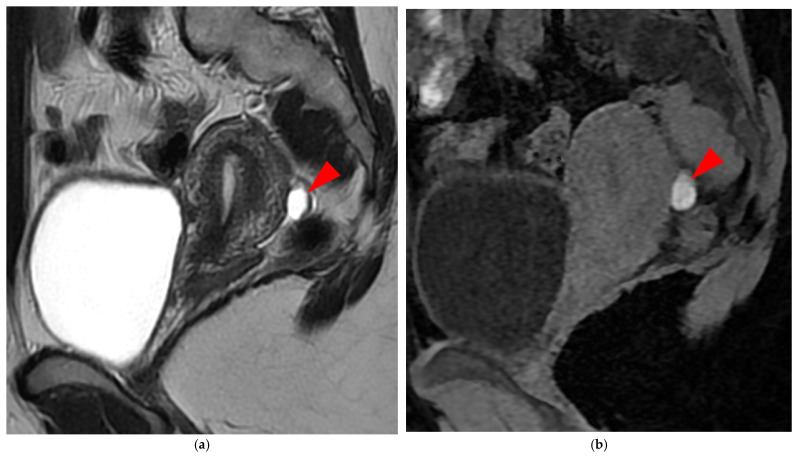
**Pelvic MRI scan of a patient with superficial endometriosis.** (**a**) Sagittal T2WI shows a hyperintense nodule located on the peritoneum of the pouch of Douglas (*arrowhead*). (**b**) Sagittal fat-suppressed T1WI reveals the hemorrhagic nature of this nodule due to its high signal intensity (*arrowhead*), allowing the diagnosis of superficial endometriosis.

**Figure 17:**
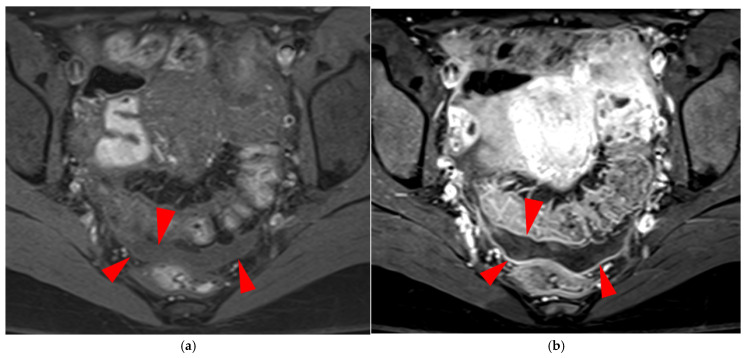
Pelvic MRI scan of a patient with likely superficial endometriosis, performed during a painful menstrual period. (**a**) Axial fat-suppressed T1WI demonstrates moderate dependent peritoneal effusion (*arrowheads*). (**b**) Axial contrast-enhanced fat-suppressed T1WI reveals perceptible curvilinear enhancement of the peritoneal layers, suggesting the possible presence of superficial endometriotic lesions (*arrowheads*).

This form of endometriosis is not listed in the HTD MRI classification, as the classification focuses exclusively on DIE primarily involving the USLs. Regarding superficial endometriosis, MRI is not a reliable diagnostic tool. However, hemorrhagic peritoneal implants appearing as hyperintensities on fat-suppressed T1WI can be detected, particularly in dependent areas (Figure 16), and correlate well with superficial endometriosis at surgery [18,23]. In our experience, an indirect sign of superficial endometriosis that may be observed during painful menstrual periods is subtle inflammation of the pelvic peritoneal layers, appearing as a regular linear enhancement on contrast-enhanced fat-suppressed T1WI (Figure 17). This sign is non-specific but could serve as a useful indicator in the clinical context of cyclic pelvic pain suggestive of endometriosis. This enhancement on MRI may occasionally be accompanied by moderate dependent peritoneal effusion.

#### 2.3.3. The Specific Case of the Retroflexed Uterus

**Figure 18:**
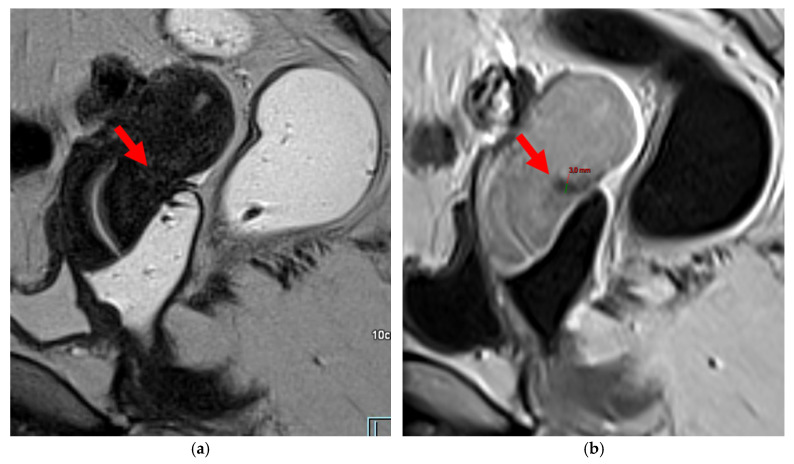
Pelvic MRI scan of a symptomatic patient with a retroflexed uterus. (**a**) Sagittal T2WI shows a uterus in a retroverted/retroflexed position, resulting in an unassessable torus uterinus area (*arrow*). (**b**) Sagittal contrast-enhanced T1WI without fat suppression highlights the thickened torus and the origins of the USLs, making them visible and measurable (*arrow*) with a thickness >2 mm.

In our experience, the injection of a contrast agent is useful in cases of uterine retroflexion. In the sagittal plane on T2WI, the torus uterinus and the origin of the USLs may be obscured by this retroflexion. In T1WI without fat suppression, the injection of a contrast agent allows for better visualization of the torus uterinus and the origin of the USLs, as these structures enhance significantly less than the adjacent myometrium due to marked proliferation of smooth muscle cells and fibrosis, thereby increasing their contrast with the adjacent myometrium (Figure 18). These structures may be considered potentially endometriotic if their thickness is >2 mm.

## 3. How to Use the HTD MRI Classification in Daily Practice

This classification is intended for use as a first-line diagnostic tool for endometriosis. Radiologists should be aware of the potentially non-specific modifications in the appearance of USLs on MRI in cases of prior pelvic surgery, peritonitis, or upper genital tract infection. These conditions can result in post-inflammatory cicatricial thickening, which may mimic or obscure endometriosis-related findings [24,25]. Awareness of these factors is essential for accurate interpretation and diagnosis.

In addition, variations in imaging protocols might affect the application and reproducibility of the HTD MRI classification.

### 3.1. Reading Strategy

The first step is to identify the USLs on T2WI in the sagittal plane, followed by the axial plane, and, if needed, the coronal plane, starting visually from the posterior aspect of the uterine cervix corresponding to the origin of the proximal USLs. Once identified, the USLs should be classified according to the HTD MRI classification. Next, signs that may warrant upgrading the USLs to a higher type should be assessed.

For linear types, verify on fat-suppressed T1WI the presence of any hemorrhagic punctum within the USLs or the torus uterinus, which would upgrade the classification to nodular type 4. Then, assess for the absence of specific visceral involvement (e.g., rectal or sigmoid, urinary, or nerve structures) that would automatically classify the USLs as nodular type 6, regardless of their appearance.

The MRI report should include the PPV and FPR associated with the highest type identified using the HTD MRI classification, providing the referring physician and the patient with clear information regarding the probabilistic nature of the MRI diagnosis.

### 3.2. The “L-Category” (Linear Types)

The grading from 1 to 3B follows the logic of a Likert scale, expressing a perceived degree of risk in a subjective and progressive manner.

(a). For types 1 and 2, despite the seemingly “normal” MRI findings, endometriosis remains possible in women with evocative clinical symptoms.
(b). For type 3A, the presence of DIE lesions is equivocal and may indicate mild involvement of the USLs.
(c). For type 3B, the presence of DIE lesions is likely and may correspond to moderate involvement of the USLs.

According to the seminal article [19], additional signs to improve diagnostic confidence for linear types 1–3 include, for example:

- Thickening of the torus uterinus (>2 mm) with hypointensity visible on sagittal T2WI, or on sagittal post-contrast T1WI without fat suppression in cases of uterine retroflexion.
- Ovarian endometriomas appearing as hyperintense lesions on T1WI with fat saturation, whose presence on MRI enhances diagnostic confidence in favor of USL endometriosis.

### 3.3. The “N-Category” (Nodular Types)

For all nodular types (4–6), the presence of DIE lesions is certain, though the severity of the disease should be evaluated using other existing classifications.

To date, only a few imaging classifications have been proposed to stage the extent of endometriosis. Examples include the rASRM, ENZIAN, Endo-Stage MRI, and the Deep Pelvic Endometriosis Index classifications [26,27,28,29]. These classifications were developed in expert centers for endometriosis, where patients typically present with more advanced stages of the disease. As a result, they are better suited for hospital radiology, advanced endometriosis cases, and pre-surgical mapping. They are also valuable for assessing surgical complication risks and fertility outcomes. However, they are often too complex and impractical for use in first-line radiology practices, where patients are more likely to present with less advanced stages of the disease, and the incidence of endometriosis differs from that seen in expert centers. Additionally, these classifications are not easily understandable for patients.

In other words, the HTD MRI classification is intended primarily for screening and routine detection (pre-referral) and may fit into more complex staging (e.g., ENZIAN) once a patient is referred to a specialized center. There, clinicians may use recognized systems (rASRM, ENZIAN, etc.) for surgical planning; however, these systems may be too detailed or cumbersome for first-line radiology practice. Hence, the HTD MRI classification complements rather than potentially supersedes existing frameworks.

## 4. Role of the HTD MRI Classification in the Diagnostic Approach

Long misunderstood and poorly diagnosed, endometriosis is now increasingly recognized by both the general public and healthcare professionals (gynecologists, midwives, general practitioners). Women suffering from this condition require rapid and targeted care to help alleviate their pain. Therefore, a structured care pathway is necessary. The integration of a patient into such a pathway should begin with a clinical evaluation, which may warrant a pelvic MRI. Some biomarkers, including the microRNA signature for endometriosis, have emerged as diagnostic tools [30,31]. Endotest^®^, for instance, is a salivary test capable of providing a rapid diagnosis within approximately one week. It is extremely reliable, allowing for early testing and prompt management of the disease. Currently, the diagnosis of endometriosis is made on average 7 to 10 years after the onset of symptoms, which is unacceptably long [32]. Diagnostic strategies aimed at shortening delays in screening and reducing medical wandering are urgently needed [33].

**Figure 19:**
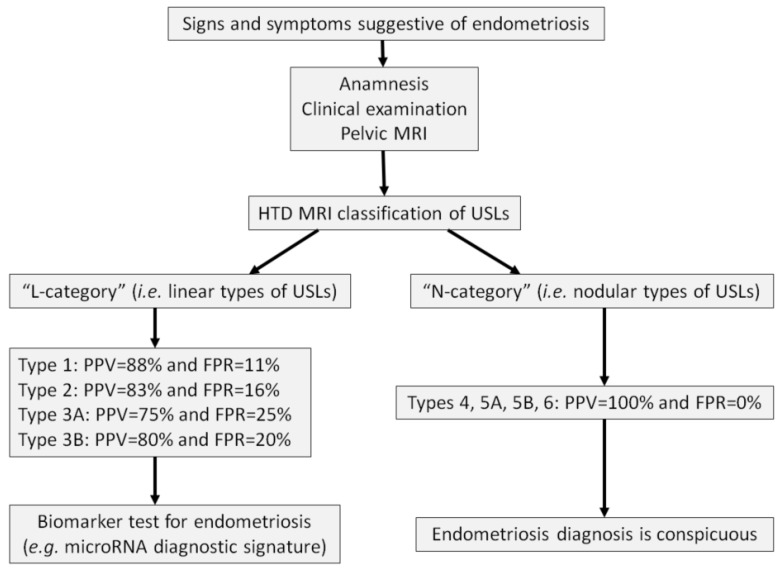
Diagnostic process for endometriosis involving clinical examination, imaging, and biomarkers. FPR: false positive rate; MRI: magnetic resonance imaging; PPV: positive predictive value; USLs: uterosacral ligaments.

From a pragmatic standpoint, an “integrative” diagnostic strategy for the initial diagnosis of endometriosis in primary care can be developed, with its cost-effectiveness already studied by Ferrier et al. [34]. The following three-step approach can be proposed (Figure 19):

(a). Identifying a patient with chronic pelvic pain suggestive of endometriosis during the clinical examination, possibly using standardized questionnaires [35].
(b). Performing a pelvic MRI to search for ovarian endometriomas or signs of DIE.
(c). Measuring biomarkers such as the salivary microRNA diagnostic signature if the MRI is interpreted as “normal”.

To optimize the cost-effectiveness of this highly sensitive and specific biotechnology, access to microRNA testing should be filtered by clinical examination followed by imaging. This approach is logical, as microRNA analysis is unnecessary if the MRI is typical of endometriosis. The perception of normality when interpreting a pelvic MRI prescribed for the evaluation of endometriosis is still often based on vague and subjective judgment. The possibility of standardization using semi-objective criteria provided by the HTD MRI classification could optimize the approach by reserving salivary microRNA analysis only for patients whose MRI shows USL types 1, 2, or 3 (PPV of endometriosis between 75% and 88%, and FPR between 11% and 25%). Patients whose MRI shows USL types 4, 5, or 6 have a PPV of endometriosis of 100%, thus not requiring microRNA confirmation.

Further studies are necessary to evaluate the robustness of this “integrative” diagnostic strategy before it can be implemented in routine clinical practice, once the measurement of specific biomarkers is definitively validated and made available.

## 5. Conclusions

MRI is widely regarded as the optimal imaging modality for diagnosing endometriosis. This pictorial essay offers radiologists a comprehensive imaging atlas, illustrating the application of the HTD MRI classification of USLs. It provides standardized interpretations supported by detailed examples for each classification type.

The HTD MRI classification serves as a valuable and sensitive screening tool in outpatient radiology for symptomatic women, contributing to a reduction in diagnostic delays associated with endometriosis. This imaging-based system has the potential to foster uniform reporting among physicians, thereby facilitating more effective patient selection for personalized diagnostic and therapeutic management.

Furthermore, the HTD MRI classification could form the foundation for developing artificial intelligence algorithms to enable automated diagnosis in radiology practices. The quantified diagnostic suspicion of endometriosis, based on the PPV associated with each USL MRI type, could also be corroborated through biomarker assessments, such as salivary microRNA signatures. This biological confirmation is particularly relevant for linear USL types, where the MRI PPV is below 100%, and the FPR remains relatively high.

## Data Availability

The data used to support the findings of this study are available from the corresponding author upon request in order to protect patient privacy.

## Abbreviations

DIE	Deep infiltrating endometriosis
FPR	False positive rate
HTD	Hôtel-Dieu
MRI	Magnetic resonance imaging
PPV	Positive predictive value
T1WI	T1-weighted imaging
T2WI	T2-weighted imaging
USL	Uterosacral ligament

## References

[1] Clement M.D., Kurman R.J. (2002). Diseases of the peritoneum (including endometriosis). Blaustein’s Pathology of the Female Genital Tract.

[2] Giudice L.C. (2010). Clinical practice. Endometriosis. N. Engl. J. Med..

[3] Chapron C., Dubuisson J.-B., Pansini V., Vieira M., Fauconnier A., Barakat H., Dousset B. (2002). Routine clinical examination is not sufficient for diagnosing and locating deeply infiltrating endometriosis. J. Am. Assoc. Gynecol. Laparosc..

[4] Koninckx P.R., Meuleman C., Cornillie F.J., Demeyere S., Lesaffre E. (1991). Suggestive evidence that pelvic endometriosis is a progressive disease, whereas deeply infiltrating endometriosis is associated with pelvic pain. Fertil. Steril..

[5] Imperiale L., Nisolle M., Noël J.C., Fastrez M. (2023). Three Types of Endometriosis: Pathogenesis, Diagnosis and Treatment. State of the Art. J. Clin. Med..

[6] Koninckx P.R., Oosterlynck D., D’Hooghe T., Meuleman C. (1994). Deeply infiltrating endometriosis is a disease whereas mild endometriosis could be considered a non-disease. Ann. N. Y. Acad. Sci..

[7] Bazot M., Thomassin I., Hourani R., Cortez A., Darai E. (2004). Diagnostic accuracy of transvaginal sonography for deep pelvic endometriosis. Ultrasound Obstet. Gynecol..

[8] Kennedy S., Bergqvist A., Chapron C., D’Hooghe T., Dunselman G., Greb R., Hummelshoj L., Prentice A., Saridogan E. (2005). ESHRE guideline for the diagnosis and treatment of endometriosis. Hum. Reprod..

[9] Bazot M., Darai E., Hourani R., Thomassin I., Cortez A., Uzan S., Buy J.N. (2004). Deep pelvic endometriosis: MR imaging for diagnosis and prediction of extension of disease. Radiology.

[10] Loubeyre P., Petignat P., Jacob S., Egger J.F., Dubuisson J.B., Wenger J.M. (2009). Anatomic distribution of posterior deeply infiltrating endometriosis on MRI after vaginal and rectal gel opacification. Am. J. Roentgenol..

[11] Umek W.H., Morgan D.M., Ashton-Miller J.A., DeLancey J.O. (2004). Quantitative analysis of uterosacral ligament origin and insertion points by magnetic resonance imaging. Obstet. Gynecol..

[12] Sampson J.A. (1927). Peritoneal endometriosis due to menstrual dissemination of endometrial tissue into the peritoneal cavity. Am. J. Obstet. Gynecol..

[13] Chapron C., Fauconnier A., Vieira M., Barakat H., Dousset B., Pansini V., Vacher-Lavenu M.C., Dubuisson J.B. (2003). Anatomical distribution of deeply infiltrating endometriosis: Surgical implications and proposition for a classification. Hum. Reprod..

[14] Audebert A., Petousis S., Margioula-Siarkou C., Ravanos K., Prapas N., Prapas Y. (2018). Anatomic distribution of endometriosis: A reappraisal based on series of 1101 patients. Eur. J. Obstet. Gynecol. Reprod. Biol..

[15] Bazot M., Gasner A., Ballester M., Daraï E. (2011). Value of thin section oblique axial T2-weighted magnetic resonance images to assess uterosacral ligament endometriosis. Hum. Reprod..

[16] Bourgioti C., Preza O., Panourgias E., Chatoupis K., Antoniou A., Nikolaidou M., Moulopoulos L. (2017). MR imaging of endometriosis: Spectrum of disease. Diagn. Interv. Imaging.

[17] Foti P.V., Farina R., Palmucci S., Vizzini I.A.A., Libertini N., Coronella M., Spadola S., Caltabiano R., Iraci M., Basile A. (2018). Endometriosis: Clinical features, MR imaging findings and pathologic correlation. Insights Imaging.

[18] Hottat N., Larrousse C., Anaf V., Noël J.C., Matos C., Absil J., Metens T. (2009). Endometriosis: Contribution of 3.0-T pelvic MR imaging in preoperative assessment--initial results. Radiology.

[19] Hélage S., Rivière L., Buy J.N., Bordonné C., Préaux F., Just P.A., Aflak N., Rousset P., Dion É. (2024). MRI classification of uterosacral ligament involvement in endometriosis: The Hôtel-Dieu classification. Br. J. Radiol..

[20] Medeiros L.R., Rosa M.I., Silva B.R., Reis M.E., Simon C.S., Dondossola E.R., da Cunha Filho J.S. (2015). Accuracy of magnetic resonance in deeply infiltrating endometriosis: A systematic review and meta-analysis. Arch. Gynecol. Obstet..

[21] Nisenblat V., Bossuyt P.M., Farquhar C., Johnson N., Hull M.L. (2016). Imaging modalities for the non-invasive diagnosis of endometriosis. Cochrane Database Syst. Rev..

[22] Ghezzi F., Raio L., Cromi A., Duwe D.G., Beretta P., Buttarelli M., Mueller M.D. (2005). “Kissing ovaries”: A sonographic sign of moderate to severe endometriosis. Fertil. Steril..

[23] Whitaker L.H., Doust A., Stephen J., Norrie J., Cooper K., Daniels J., Hummelshoj L., Cox E., Beatty L., Chien P. (2021). Laparoscopic treatment of isolated superficial peritoneal endometriosis for managing chronic pelvic pain in women: Study protocol for a randomised controlled feasibility trial (ESPriT1). Pilot. Feasibility Stud..

[24] Delanian S. (2008). Post-operative fibrosis: Pathophysiological aspects and therapeutical perspectives. Chir. Main..

[25] Ruaux E., VanBuren W.M., Nougaret S., Gavrel M., Charlot M., Grangeon F., Bolze P.-A., Thomassin-Naggara I., Rousset P. (2024). Endometriosis MR mimickers: T2-hypointense lesions. Insights Imaging.

[26] Haas D., Shebl O., Shamiyeh A., Oppelt P. (2013). The rASRM score and the Enzian classification for endometriosis: Their strengths and weaknesses. Acta Obstet. Gynecol. Scand..

[27] Bazot M., Daraï E., Benagiano G.P., Reinhold C., Favier A., Roman H., Donnez J., Bendifallah S. (2022). ENDO_STAGE magnetic resonance imaging: Classification to screen endometriosis. J. Clin. Med..

[28] Thomassin-Naggara I., Monroc M., Chauveau B., Fauconnier A., Verpillat P., Dabi Y., Gavrel M., Bolze P.A., Darai E., Touboul C. (2023). Multicenter external validation of the Deep Pelvic Endometriosis Index magnetic resonance imaging score. JAMA Netw. Open.

[29] Harth S., Roller F.C., Zeppernick F., Meinhold-Heerlein I., Krombach G.A. (2023). Deep Infiltrating Endometriosis: Diagnostic Accuracy of Preoperative Magnetic Resonance Imaging with Respect to Morphological Criteria. Diagnostics.

[30] Moustafa S., Burn M., Mamillapalli R., Nematian S., Flores V., Taylor H.S. (2020). Accurate diagnosis of endometriosis using serum microRNAs. Am. J. Obstet. Gynecol..

[31] Bendifallah S., Suisse S., Puchar A., Delbos L., Poilblanc M., Descamps P., Golfier F., Jornea L., Bouteiller D., Touboul C. (2022). Salivary microRNA signature for diagnosis of endometriosis. J. Clin. Med..

[32] Agarwal S.K., Chapron C., Giudice L.C., Laufer M.R., Leyland N., Missmer S.A., Singh S.S., Taylor H.S. (2019). Clinical diagnosis of endometriosis: A call to action. Am. J. Obstet. Gynecol..

[33] Spiers A., Roman H., Wasson M., Chapron C., Rousset P., Golfier F., Fauvet R., Delbos L., Poilblanc M., Lavoué V. (2024). Clues to revising the conventional diagnostic algorithm for endometriosis. Int. J. Gynaecol. Obstet..

[34] Ferrier C., Bendifallah S., Suisse S., Dabi Y., Touboul C., Puchar A., Zarca K., Durand Zaleski I. (2023). Saliva microRNA signature to diagnose endometriosis: A cost-effectiveness evaluation of the Endotest^®^. BJOG.

[35] Chapron C., Lafay-Pillet M.C., Santulli P., Bourdon M., Maignien C., Gaudet-Chardonnet A., Maitrot-Mantelet L., Borghese B., Marcellin L. (2022). A new validated screening method for endometriosis diagnosis based on patient questionnaires. EClinicalMedicine.

